# Design and implementation of a portable snapshot multispectral imaging crop-growth sensor

**DOI:** 10.3389/fpls.2024.1416221

**Published:** 2024-08-26

**Authors:** Yongxian Wang, Jingwei An, Jianshuang Wu, Mingchao Shao, Jiacheng Wang, Xia Yao, Xiaohu Zhang, Chongya Jiang, Yongchao Tian, Weixing Cao, Dong Zhou, Yan Zhu

**Affiliations:** ^1^ College of Agriculture, Nanjing Agricultural University, Nanjing, China; ^2^ National Engineering and Technology Center for Information Agriculture, Ministry of Industry and Information Technology, Nanjing, China; ^3^ Engineering Research Center of Smart Agriculture, Ministry of Education, Nanjing, China; ^4^ Key Laboratory for Crop System Analysis and Decision Making, Ministry of Agriculture and Rural Affairs, Nanjing, China; ^5^ Jiangsu Key Laboratory for Information Agriculture, Jiangsu Provincial Department of Science and Technology, Nanjing, China; ^6^ Collaborative Innovation Center for Modern Crop Production Co-sponsored by Province and Ministry, Ministry of Education, Nanjing, China

**Keywords:** crop growth monitoring, portable snapshot multispectral imaging crop-growth sensor, wide band co-optical path imaging system, mosaic filter spectroscopy, field experiments, prediction models

## Abstract

The timely and accurate acquisition of crop-growth information is a prerequisite for implementing intelligent crop-growth management, and portable multispectral imaging devices offer reliable tools for monitoring field-scale crop growth. To meet the demand for obtaining crop spectra information over a wide band range and to achieve the real-time interpretation of multiple growth characteristics, we developed a novel portable snapshot multispectral imaging crop-growth sensor (PSMICGS) based on the spectral sensing of crop growth. A wide-band co-optical path imaging system utilizing mosaic filter spectroscopy combined with dichroic mirror beam separation is designed to acquire crop spectra information over a wide band range and enhance the device’s portability and integration. Additionally, a sensor information and crop growth monitoring model, coupled with a processor system based on an embedded control module, is developed to enable the real-time interpretation of the aboveground biomass (AGB) and leaf area index (LAI) of rice and wheat. Field experiments showed that the prediction models for rice AGB and LAI, constructed using the PSMICGS, had determination coefficients (R²) of 0.7 and root mean square error (RMSE) values of 1.611 t/ha and 1.051, respectively. For wheat, the AGB and LAI prediction models had R² values of 0.72 and 0.76, respectively, and RMSE values of 1.711 t/ha and 0.773, respectively. In summary, this research provides a foundational tool for monitoring field-scale crop growth, which is important for promoting high-quality and high-yield crops.

## 1 Introduction

Real-time and accurate estimations of crop growth conditions provide fundamental agricultural information for crop growth diagnosis and precise management, playing a crucial role in enhancing crop yields and quality (Karthikeyan et al., 2020; Berger et al., 2022). Traditional methods of obtaining crop growth information have relied on subjective observations by agricultural experts or destructive sampling combined with physical and chemical experiments in the laboratory. However, these methods have several disadvantages, such as their poor timeliness, time-consuming processes, and labor-intensive procedures (Zou et al., 2001; Yuan et al., 2022). In recent years, crop growth monitoring technologies based on spectral imaging have been developed; these technologies are non-destructive, provide data in real time, and are highly efficient. These technologies have found widespread applications in estimating the nitrogen content in rice leaves (Zhou et al., 2018), monitoring wheat biomass (Jia et al., 2019), and detecting powdery mildew in wheat (Xuan et al., 2022). Spectral imaging sensors, which can serve as an implementation platform for spectral imaging technology, are foundational tools for crop growth monitoring (Sun et al., 2022).

Agricultural scientists demand crop growth monitoring at various scales, and different types of spectral imaging sensing devices offer possibilities for fulfilling this need. Portable spectral imaging devices, characterized by their portability and ease of operation, have demonstrated significant advantages in acquiring information on crop organs and canopies at the field scale (Pallottino et al., 2019; Kim et al., 2023). Jia et al. (2019) utilized a commercial hyperspectral imaging device, the GaiaField-V10E (400−1000 nm), to estimate wheat leaf biomass. They used the synergistic interval partial least squares (SIPLS) and successive projection algorithm (SPA) to select eight feature wavelengths and construct a wheat leaf biomass prediction model based on partial least squares regression (PLSR) (R²=0.79, RMSE=0.059 kg/m^2^). While this type of device enables rich spectral information acquisition, data processing relies on specialized remote sensing personnel, and it cannot directly output crop growth information. Shao et al. (2023) used a portable pole-mounted commercial multispectral camera, RedEdge, for acquiring multispectral images of maize. After offline cropping, registration, and radiometric correction of the obtained images, they achieved maize leaf area index (LAI) prediction (accuracy R²=0.816, RMSE=0.399). However, the multi-channel multispectral camera used in this study was affected by field-of-view differences between lenses in near-ground applications, and the process from crop spectral information acquisition to agricultural parameter interpretation relied on a multi-step offline processing approach.

In comparison with commercial spectral imaging devices, some research institutions have developed agriculture-specific spectral imaging devices. Wang et al. (2020b) designed a handheld corn hyperspectral imaging system that included a commercial hyperspectral camera, a leaf scanner, a lightbox, and a controller. Using the normalized difference vegetation index (NDVI) combined with PLSR, they constructed models to predict corn leaf nitrogen content and relative water content. However, the device is relatively large, and efficient imaging requires moving the hyperspectral camera for the push-broom acquisition of corn leaf images. The development of prediction models for multiple crop growth parameters is still ongoing. Tang et al. (2022) developed a portable wheat chlorophyll detector using a commercial mosaic multispectral camera (700−900 nm) with 25 bands. This instrument, which comprised a spectral camera, a control module, and a network module, could construct a wheat chlorophyll content prediction model by selecting optimal feature bands. Although this device provided direct interpretation of wheat chlorophyll, its bands were concentrated in the near-infrared and red-edge spectra, offering a limited wavelength range, and the output indicators were relatively singular. Wang et al. (2020a) also created a portable soybean leaf multispectral imaging device consisting of a monochrome camera, different wavelength light-emitting diodes (LEDs), and a controller. This device captured multispectral images of soybeans by pressing the soybeans flat and then constructed NDVI images. However, research findings did not present a prediction model specifically for soybean chlorophyll content.

To address the aforementioned typical issues, this study introduces a novel portable snapshot multispectral imaging crop growth sensor (PSMICGS) capable of real-time interpretation of rice and wheat aboveground biomass (AGB) and leaf area index (LAI). In contrast to previous work, this study offers the following significant contributions:

1. A design approach for PSMICGS using mosaic filters (MFs) in conjunction with dichroic mirrors (DMs) to achieve wide-band integrated co-optical imaging is proposed. This method enables the real-time acquisition, processing, and interpretation of crop spectral information across a broad wavelength range.
2. Assembly and adjustment methods for a wide-band co-optical front imaging system incorporating DMs for beam separation were explored, as well as methods for the registration of multispectral images. This method included strategies for real-time online image registration and multispectral image fusion, enhancing capabilities for crop analysis.
3. A processor system that integrated sensor data with crop growth monitoring models based on an embedded control module was developed. This addressed the limitation of existing devices that struggle with real-time crop growth interpretation. Field experiments were conducted in rice and wheat fields using the PSMICGS, resulting in the construction of prediction models for rice and wheat AGB and LAI.

## 2 Materials and methods

In this section, we detail the design process of the PSMICGS and the construction of the models for estimating AGB and LAI in rice and wheat fields. Specifically, we outline the design approach of a wide-band co-optical path imaging system based on MFs combined with DMs for spectral separation. Additionally, we describe the experimental design for monitoring AGB and LAI in rice and wheat using the PSMICGS, along with the process used to construct prediction models.

### 2.1 Design of the PSMICGS for wide band integrated co-optical path imaging

#### 2.1.1 Selection of crop growth-sensitive spectral bands

After solar radiation interacts with crops, spectral information is formed through absorption, transmission, and reflection. This information reflects canopy structure, growth conditions, and physiological and biochemical characteristics of crops, with reflection spectra being commonly used in crop growth monitoring (Xue et al., 2003; Zhu et al., 2007a). To develop a PSMICGS capable of direct crop growth interpretation, it is necessary to carefully select crop growth-sensitive bands. Based on our unit’s research on crop growth monitoring (Zhu et al., 2007b; Wang et al., 2012; Li et al., 2022) and diagnostic equipment development (Ni et al., 2018; Yao et al., 2020; Yuan et al., 2022) at the National Engineering and Technology Center for Information Agriculture, Nanjing Agricultural University, China, it was found that canopy reflectance in rice and wheat is closely related to specific spectral ranges: 413–434 nm, 517–538 nm, 553–577 nm, 660–680 nm, and 700–770 nm for nitrogen content (Wang et al., 2012; Yao et al., 2013); 706–738 nm and 806–816 nm for biomass (Yao et al., 2018; Jia et al., 2019); and 590–710 nm and 745–1130 nm for wheat leaf dry weight and LAI (Feng et al., 2009). Considering these studies, we selected 458, 487, 527, 558, 644, 716, 737, and 813 nm as characteristic bands for the PSMICGS.

#### 2.1.2 Design of the PSMICGS control system

**Figure 1:**
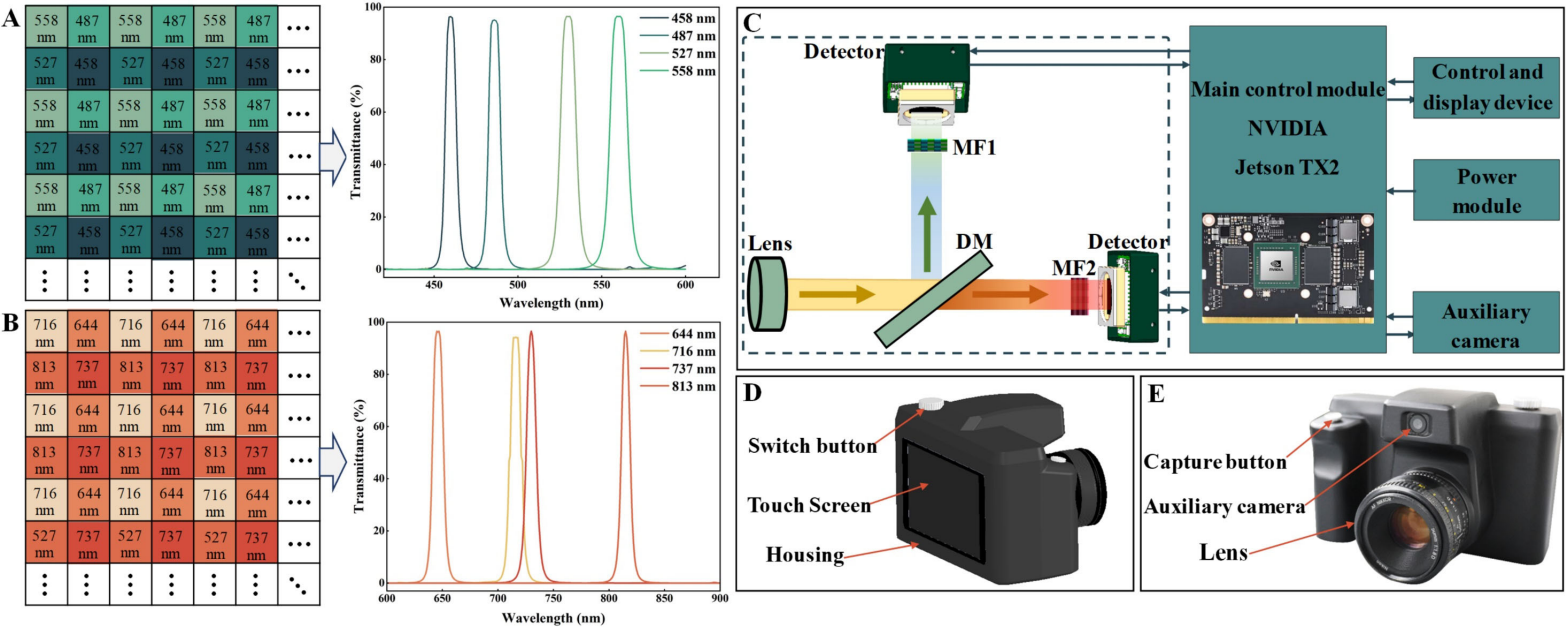
Portable snapshot multispectral imaging crop growth sensor (PSMICGS) hardware system. **(A)** MF1 band settings and transmittance curves for each band. **(B)** MF2 band settings and transmittance curves of each band. **(C)** Schematic diagram of the PSMICGS hardware system architecture. **(D)** Three-dimensional model of the entire PSMICGS machine structure. **(E)** Physical diagram of the entire PSMICGS machine structure.

The hardware architecture of the PSMICGS consisted of a front imaging system, primary control module, auxiliary cameras, power module, and control display. The front imaging system, which is crucial for capturing crop information across different spectral bands integrated a lens (AF Nikkor 50 mm F/1.8D, Tochigi Nikon Precision Co., Ltd., Japan) with a minimum focusing distance of 0.45 meters and a diagonal field of view of 46°. It utilized DMs for spectral separation and two mosaic multispectral cameras (MMC1 and MMC2) equipped with mosaic filters (MF1 and MF2). To capture crop light information at preset wavelengths, we used MF for spectral splitting. This spectral splitting technique can deposit different bands on the same mosaic template. However, due to manufacturing constraints, the eight preset bands were divided between MF1 and MF2, each fabricated using a narrowband Fabry-Pérot microcavity array method to ensure over 95% light transmittance at each central wavelength. **Figures 1A, B** show the specific band settings and transmittance curves, with MF1 containing the first four and MF2 the last four of the selected characteristic bands.

The MMC1 and MMC2 detectors were complementary metal oxide semiconductor (CMOS) image sensors with a spectral response range of 400−1000 nm, used for capturing and converting crop light information. To minimize field of view differences, a DM was employed to separate crop light information, with a reflecting wavelength range of 380−580 nm and transmitting wavelength range of 610−880 nm. The DM was positioned at a 45° angle in the system (**Figure 1C**). The primary control module of the PSMICGS utilized an NVIDIA Jetson TX2 (NVIDIA Corporation, USA), which was responsible for the real-time control of MMCs, and auxiliary cameras to collect and process crop spectral information. Communication and power supply to MMC and the auxiliary camera were facilitated using universal serial bus (USB) 3.0 and USB 2.0 technology, respectively. The auxiliary camera, a five-megapixel driver-free module, captured crop red-green-blue (RGB) images and monitored sensor field of view status in real-time. A 14.8-V lithium battery powered the primary control module, while a 5.5-inch capacitive touchscreen served as the control display for interactive sensor information processing. Data storage utilized a 256-GB high-speed TransFlash (TF) memory card (Western Digital Corporation, USA) inserted into the Secure Digital (SD) card slot on the development board. **Figures 1D, E** depict the three-dimensional model and physical appearance of the PSMICGS, respectively, with a black coating applied to the housing to minimize external light interference.

**Figure 2:**
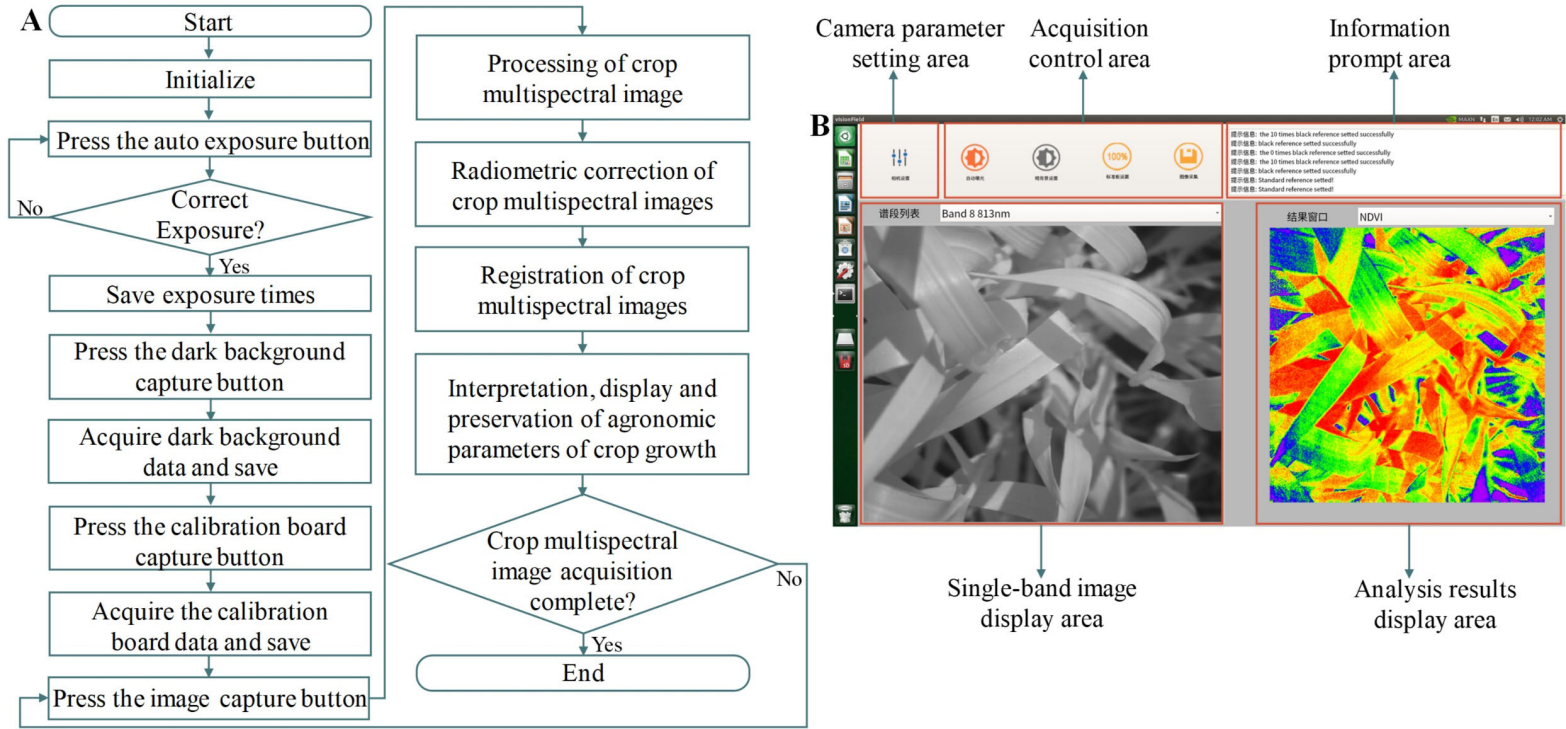
PSMICGS software system. **(A)** Workflow of the software system and **(B)** diagram of the software interface.

The PSMICGS software system was developed using the Qt development platform in conjunction with the detector software development toolkit (**Figure 2**). The software workflow is illustrated in **Figure 2A** and comprises three primary steps: initial sensor setup, spectral image acquisition and processing, and data storage. **Figure 2B** shows the graphical user interface (GUI), featuring five main sections: camera parameter settings, acquisition control, information prompts, single-band image display, and analysis result display.

### 2.2 Research on spectral image processing methods of the PSMICGS

**Figure 3:**
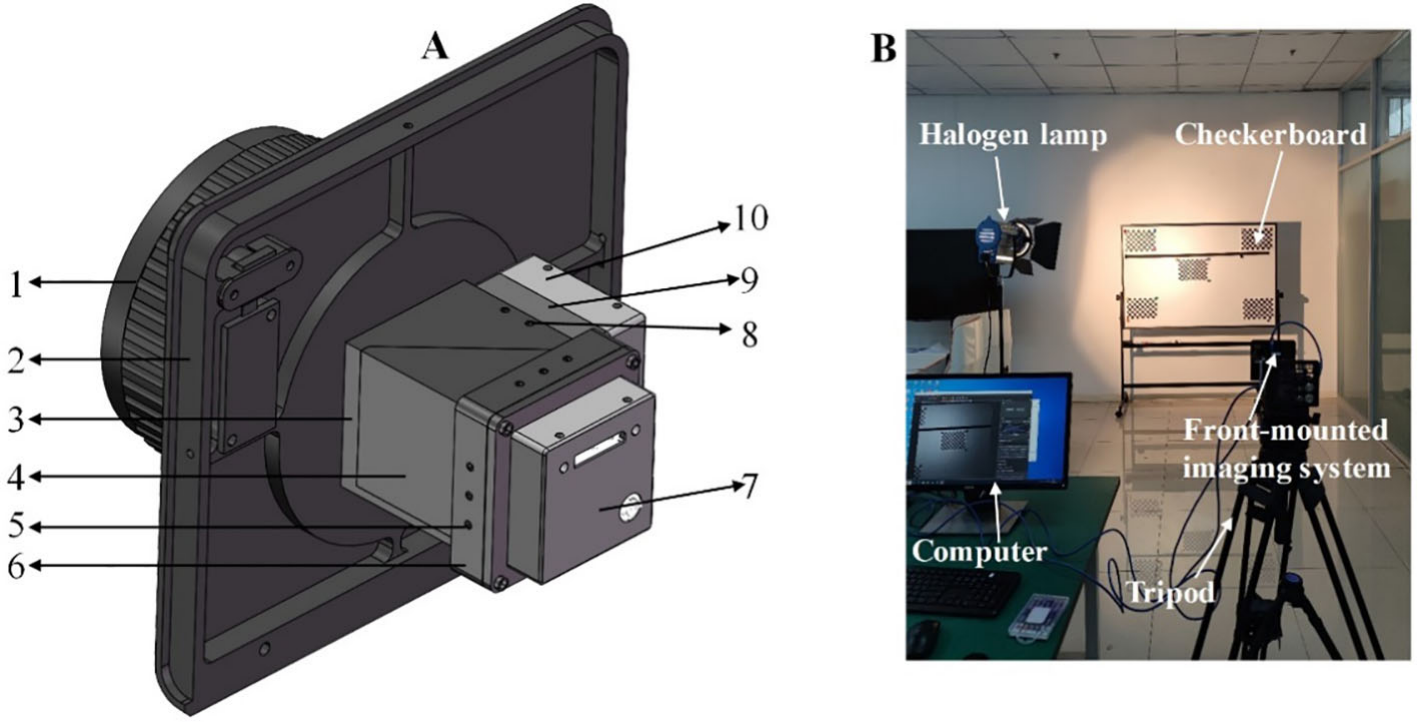
Assembly and adjustment of the PSMICGS. **(A)** Three-dimensional diagram of the front imaging system: 1. Lens, 2. Front panel, 3. Component fixing device, 4. DM and mounting fixing device, 5. Second hexagon socket set screws with cup point, 6. Second fixing adjustment piece, 7. MMC2, 8. First hexagon socket set screws with cup point, 9. First fixing adjustment piece, and 10. MMC1. **(B)** On-site image of the front imaging system assembly and adjustment.

A dual-detector integrated co-optical path imaging system was designed to achieve real-time acquisition of wide-band crop spectral information and improve device miniaturization and integration, as illustrated in **Figure 3A**. However, achieving precise alignment of the field of view for the two cameras posed challenges due to machining tolerances in mechanical components and assembly errors, and required calibration. **Figure 3B** shows the system calibration process, which involved using black-and-white checkerboard patterns. Initially, MMC1 and MMC2 were focused by adjusting them to clearly display registration reference lines, checkerboard patterns, and hybrid images. Subsequently, we adjusted MMC2 laterally or longitudinally using the second fixed adjusting piece’s square slot and second hexagon socket set screws with cup points until the registration reference line and checkerboard patterns were aligned both horizontally and vertically in their respective real-time views. MMC1 was then rotated using the circular slot of the first fixed adjustment component until the checkerboard patterns were perfectly aligned along the edge direction, and it was then secured with the first hexagon socket set screws with cup points. Finally, fine-tuning of MMC1 and MMC2 was conducted using the first and second hexagon socket set screws with cup points until the checkerboard patterns in all directions were aligned, thereby concluding the assembly and adjustment process of the front imaging system.

Upon completing the assembly and adjustment of the front imaging system, we utilized the scale-invariant feature transform (SIFT) algorithm for image registration between the two fields to further enhance co-field of view imaging accuracy (Lowe, 2004). Additionally, an affine transformation was applied for image fusion. For the specific application scenario, images were captured at a height of 70 cm above the canopy. Post-registration, fusion and the root mean square error (RMSE) were employed to assess registration accuracy, as expressed in Equation 1. If the RMSE value exceeded four, the image registration was deemed unsuccessful (Gong et al., 2013; Ma et al., 2016). To distinguish it from RMSE below, we used the RMSEr here to denote the RMSE.


(1) RMSEr=1m∑i=1m(xi−xi')2−(yi−yi')2

where *m* represents the total number of pixels and $\left(x_{i},y_{i}\right)$ and (xi',yi') represent the pixel coordinates of the reference image and the image to be registered, respectively.

### 2.3 Research on calibration methods for the PSMICGS

#### 2.3.1 Spectral calibration of the PSMICGS

**Figure 4:**
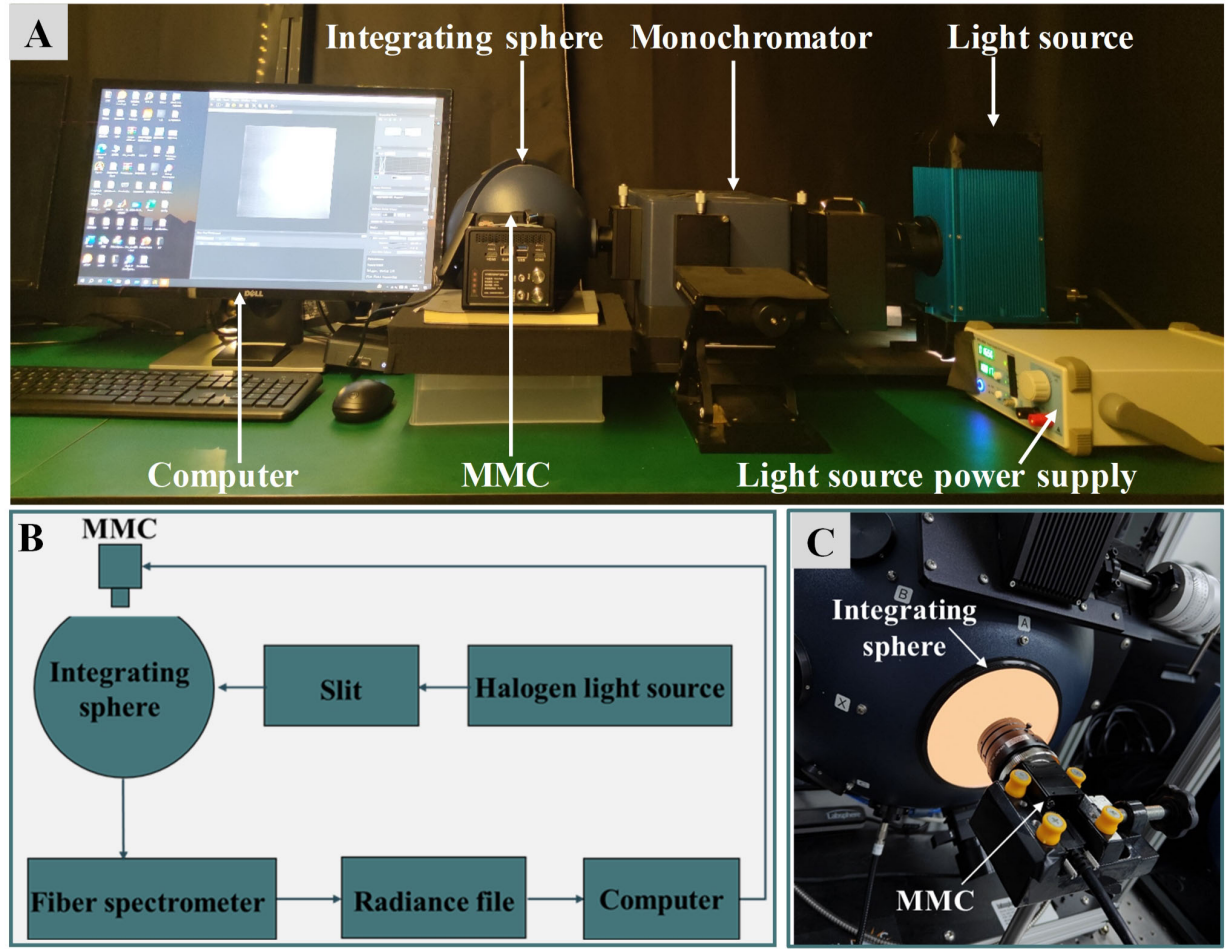
Spectral and radiometric calibration system of the PSMICGS. **(A)** On-site image of the spectral calibration. **(B)** System block diagram of the radiation calibration. **(C)** Site diagram of the setup for radiation calibration.

To characterize the response of each spectral channel of the PSMICGS, we used a spectral calibration system based on an adjustable monochromatic light source (Zolix Instruments Co., Ltd., China). As shown in **Figure 4A**, this system was composed of an integrating sphere, an adjustable monochromatic light source, a spectrometer, and a computer. The monochromatic light source covered a spectral range of 350−1000 nm, which was sufficient to cover all bands used by the PSMICGS in this study. During calibration, the sensor lens was initially aligned with the integrating sphere’s light port. The exposure time and gain of the MMC were set to 100 ms and 6 dB, respectively. The monochromator was adjusted to each of the eight MF bands to determine the band with the highest digital number (DN) response, which served as the reference band. Using this reference band as a benchmark, the monochromator was tuned to corresponding bands. When the DN reached approximately 60−80% of its maximum value, the exposure time and gain settings were recorded as benchmark parameters. Subsequently, the spectrometer bands of the monochromatic light source system were adjusted in 2-nm increments, and internal images of the integrating sphere were captured and stored for each increment. The wavelength adjustment ranges for MMC1 and MMC2 were 350−700 nm and 500−900 nm, respectively. Finally, DN values corresponding to each band were extracted based on MMC band settings, followed by fitting analysis.

#### 2.3.2 Radiometric calibration of the PSMICGS

Since the original pixel values obtained by the PSMICGS were in DN (a dimensionless unit), it was necessary to convert these values into meaningful radiance or reflectance units for interpreting crop growth parameters. Thus, we investigated the linear relationship between sensor DN and radiance, which is crucial for improving the quality and accuracy of crop multispectral images. The radiometric calibration system used in this study is illustrated in **Figures 4B, C**, and it primarily consisted of a 4-inch aperture integrating sphere (USLR-V12F-NMNN, Labsphere Inc., USA). The spectrometers used in the system were the Maya2000 Pro, with a spectral range of 165−1100 nm, and the NIRQuest, with a range of 900−1700 nm (Ocean Optics, USA). In the specific calibration procedure, four exposure time levels were set: 60, 80, 100, and 120 ms. For each exposure time, seven radiance levels were adjusted, and ten images were acquired for each radiance level. The average DN values of these images were calculated, and the DN values corresponding to each band were extracted for the fitting analysis.

### 2.4 Design of the performance test experiment based on the PSMICGS

#### 2.4.1 Signal-to-noise ratio testing experiment

The signal-to-noise ratio (SNR) is a key indicator for assessing the performance of spectral imaging sensors, and a high image SNR is a prerequisite for effective crop-growth monitoring (Li et al., 2023). In this study, we used an adjustable monochromatic light source system (**Figure 4A**) to capture images of a uniform surface light source. Initially, we set the exposure time and gain values determined during the spectral calibration process as benchmark parameters. Subsequently, the monochromator was set to 0 nm, and the exposure time and gain values of the MMC were adjusted to these benchmark parameters to avoid image overexposure. The adjustment was then halted, and the MMC captured 10 images within the integrating sphere. Finally, pixel values from the corresponding channels were extracted based on the defined positions of the reference bands on the MF, and the SNR was calculated using the following formula (Zaunseder et al., 2022):


(2) $$SNR\left(\lambda \right)=\frac{D(\lambda )}{N}=\frac{\frac{1}{n}\sum D_{i}\left(\lambda \right)}{\sqrt{\frac{{\sum \left[D_{i}\left(\lambda \right)-\frac{1}{n}\sum D_{i}\left(\lambda \right)\right]}^{2}}{n-1}}},$$

where D(λ) represents the mean value of the DN from multiple acquired spectral images, indicating the signal value, and *N* represents the RMSE of the DN from multiple acquired spectral images, representing the noise value.

#### 2.4.2 Radiometric response accuracy testing experiment

**Figure 5:**
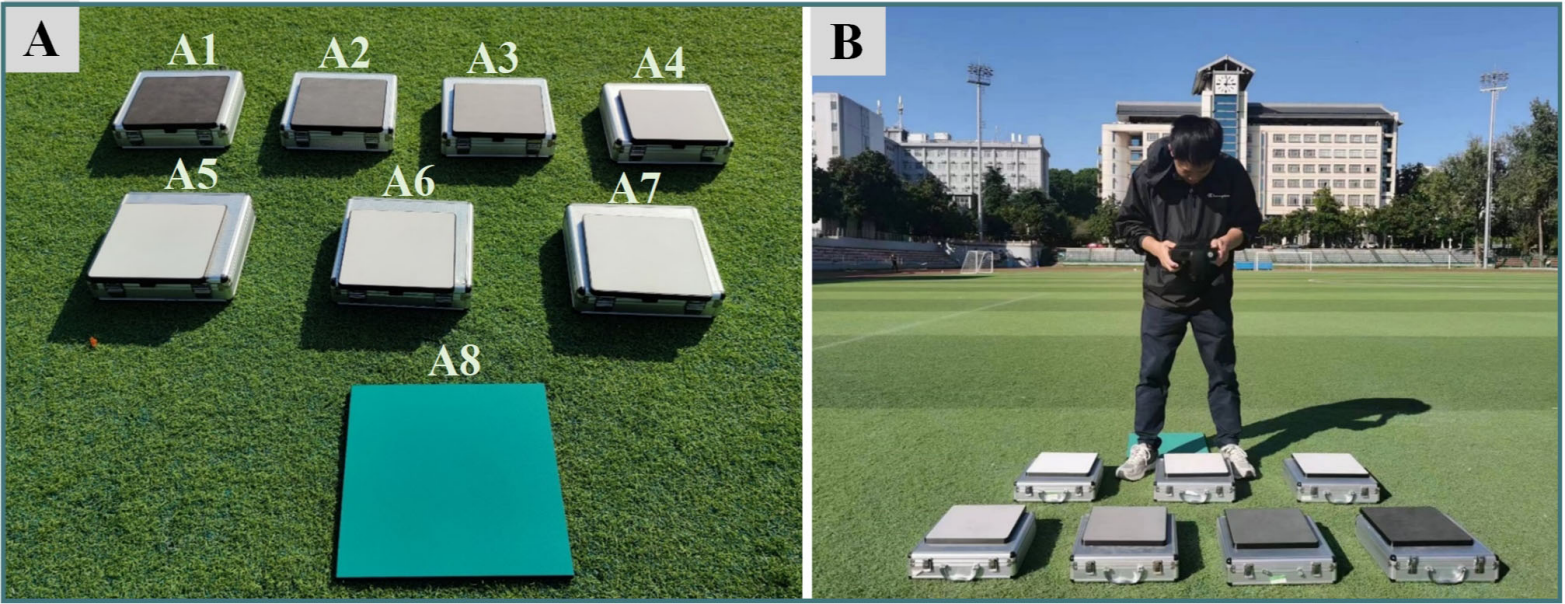
Radiometric response accuracy test of the PSMICGS. **(A)** Standard diffuse reflective panels with varying reflectivities, and **(B)** test site.

Accurate radiometric response is essential for quantitative monitoring of crop growth using the PSMICGS. We tested the PSMICGS with eight standard diffuse reflectance panels: A1−A7 (Labsphere Inc., North Sutton, NH, USA) and A8 (Changhui Electronic Technology Co., Ltd., China) (**Figures 5A, B**). The reflectance values of panels A1−A7 were 5%, 10%, 20%, 40%, 60%, 75%, and 99%, respectively, while the reflectance of panel A8 ranged from 20% to 30%.

During the experiment, the hyperspectral sensor ASD FieldSpec 4 (Analytical Spectral Devices, Boulder, CO, USA) was used to measure the actual values of each panel, with the values obtained using the PSMICGS considered as the test values. The distance between the PSMICGS device and each panel was maintained at 70 cm during data collection, and image reconstruction by the PSMICGS used a bicubic interpolation algorithm (Keys, 1981). Due to an integration gap between the MF and the detector, crosstalk between different bands can occur, affecting data accuracy. Therefore, correcting crosstalk in each reconstructed band image is fundamental for sensor data analysis. In this study, spectral crosstalk correction was achieved using Equation 3, which corrected crosstalk information within each macro-pixel region using a linear combination of crosstalk correction coefficients, $C_{ij}$ (provided by the MMC manufacturer; see the **Supplementary Material**) and the original pixel response values, $PO_{j}$ .


(3) $$\begin{array}{c} {\text{P}}_{i}=\sum_{j=1}^{n}C_{ij}\cdot PO_{j} \end{array}$$

where ${\text{P}}_{i}$ is the crosstalk-corrected response value; PO is the original response value within the macro-pixel region before crosstalk correction; $C_{ij}$ is the crosstalk correction coefficient matrix; and *i* and *j* are the pixel indices within the reconstructed and original macro-pixel regions, respectively.

To control for errors, both the ASD and PSMICGS underwent radiometric calibration using panel A7 with a reflectance of 99% prior to actual testing. The reflective values of the target measured using the PSMICGS were calculated using Equation 4. Post data collection, ENVI 5.3 software (Environment for Visualizing Images, Research Systems Inc., Boulder, CO, USA) was used to select regions of interest (ROI) to obtain the reflectance values of each panel. Due to inconsistent wavelength ranges and spectral resolutions between the two devices, Equation 5 was employed to convert the ASD test data to equivalent PSMICGS data, as suggested by Luo et al. (2023). Finally, by analyzing the test values of each panel, relative error values were calculated using Equation 6.


(4) $$R=\frac{D_{r}-D_{d}}{D_{w}-D_{d}}*R_{w}$$

where *R* is the target reflectance value; $D_{r}$ , $D_{d}$ , and $D_{w}$ are the image DNs of the target, the dark background (obtained using the lens cap to cover the lens), and the 99% reflectance calibration plate, respectively; and $R_{w}$ is the reflectance value of the 99% reflectance plate, set to 0.99 for this calculation.


(5) $$R_{r}\left(b_{i}\right)=\frac{\int_{l_{1}}^{l_{2}}R_{r}\left(l\right)SRF\left(l\right)d\left(l\right)}{\int_{l_{1}}^{l_{2}}SRF\left(l\right)d\left(l\right)}$$

where $R_{r}\left(b_{i}\right)$ is the equivalent reflectance of the i-th band; $l_{1}$ and $l_{2}$ are the band ranges, which here are 350−700 and 500−900 nm, respectively; $R_{r}$ ( l ) is the spectral reflectance of the target panels measured by the ASD; and SRF(l) is the spectral response function at wavelength *l* (obtained using through spectral calibration).


(6) $$\text{δ}=\frac{\left|x-D\right|}{D}*100\%$$

where *x* is the measured value; *D* is the true value; and δ denotes the relative error.

### 2.5 Rice and wheat growth monitoring experiment based on the PSMICGS

#### 2.5.1 Experimental design

**Figure 6:**
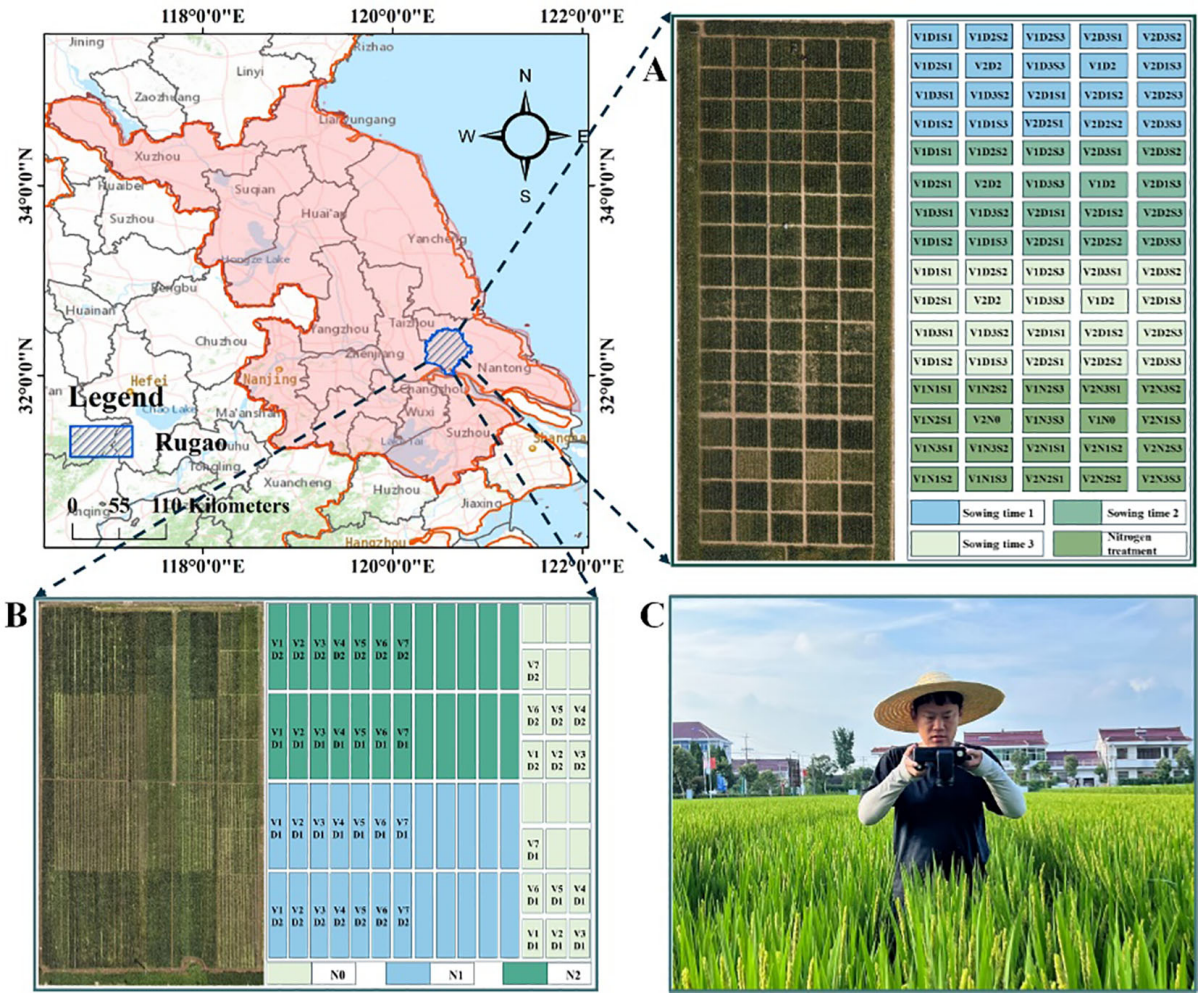
Research location and field experimental layout. **(A)** Layout of the wheat trial site. **(B)** Layout of the rice trial site. **(C)** Layout diagram of the PSMICGS collection of crop spectral images.

The wheat growth monitoring experiment was conducted from March to May 2023 at the Baipu Experimental Station in Rugao City, Jiangsu Province, China (32°26′N, 120°75′E) (**Figure 6A**). The experiment included three sowing periods: October 25, 2022 (sowing period 1), November 10, 2023 (sowing period 2), and November 25, 2023 (sowing period 3). The wheat varieties used were V1 (Yangmai 23) and V2 (Jimai 22). Four nitrogen levels were applied: N0 (0 kg/ha), N1 (52 kg/ha), N2 (104 kg/ha), and N3 (156 kg/ha). Three seeding densities were implemented: D1 (125 plants/m^2^), D2 (225 plants/m^2^), and D3 (325 plants/m^2^), with a row spacing of 30 cm. Each treatment had three replicates. Wheat was sown manually in rows, totaling 80 plots, with each plot covering an area of 18 m^2^ (4.5 m × 4 m). Spectral images and agronomic parameters were simultaneously collected during the tillering, jointing, and booting stages of wheat growth, and the total sample size was 190.

The rice growth monitoring experiment was conducted from July to September 2023 at the Baipu Experimental Station in Rugao City, Jiangsu Province, China (32°26′N, 120°75′E) (**Figure 6B**). The experiment used seven rice varieties: V1 (Yingxiang 1), V2 (Nanjing Yinggu), V3 (Taixiangjing 1402), V4 (Sidao 20), V5 (Yangxiangyu 1), V6 (Sidao 17), and V7 (Yanjing 23). Three nitrogen levels were considered: N0 (0 kg/ha), N1 (150 kg/ha), and N2 (300 kg/ha). Two planting row spacings were used: D1 (60 cm) and D2 (30 cm). Rice planting was conducted using a machine transplanting method, totaling 72 plots. Spectral images and agronomic parameters were simultaneously collected during the rice jointing, booting, and heading stages of the rice growth, and the total sample size was 126.

#### 2.5.2 Test equipment and methods

After assembling the hardware system of the PSMICGS as designed in this study and finalizing the software packages, the PSMICGS was comprehensively prepared for field growth monitoring of rice and wheat (**Figure 6C**). To obtain crop reflectance values, radiometric calibration was performed using a 40% reflectance Lambertian diffuser prior to the experiment. The reflectance was then calculated online using the control module in the system combined with Equation 3, where $R_{w}$ was set to 0.4. During the collection of rice and wheat multispectral images, the PSMICGS was positioned at a height of 70 cm from the canopy, and data collection was conducted under clear weather conditions between 10:00 a.m. and 2:00 p.m.

To obtain the required agronomic parameters for rice and wheat, in the wheat experiment, five representative wheat plants were selected from each plot. In the rice experiment, three representative rice plants were selected from each plot. The rice and wheat samples were then separated into stems, leaves, and panicles. The Li–3000c leaf area meter (Li–Cor., Lincoln, NE, USA) was used to measure the leaf area of the rice and wheat samples, and the LAI of the population was calculated by multiplying the number of plants by the tillers per square meter. After dissection, the plants were placed in an oven, blanched at 105°C for half an hour, dried at 80°C to a constant weight, and finally, the AGB per unit land area was calculated based on the sampled area.

#### 2.5.3 Multispectral image processing and VI selection

Following the acquisition of crop multispectral image, the software ENVI was utilized for selecting ROI. The entire field of view was designated as the ROI, and the reflectance values for rice and wheat were averaged from these areas. Vegetation indices (VIs) were computed using Matlab2021 (The MathWorks, Natick, MA, USA). To achieve quantitative monitoring of rice and wheat AGB and LAI, this study calculated six VIs based on the extracted canopy reflectance, as shown in **Table 1**. These selected VIs have been widely used in previous studies focused on monitoring rice and wheat growth.

**Table 1:** VIs for the LAI and AGB estimations in rice and wheat.

VI	Name	Formulation	Reference
GNDVI	Green normalized difference vegetation index	$\frac{NIR-G}{NIR+G}$	(Gitelson et al., 1996)
NDVI	Normalized difference vegetation index	$\frac{NIR-R}{NIR+R}$	(Candiago et al., 2015)
NDRE	Normalized difference red-edge	$\frac{NIR-RE}{NIR+RE}$	(Fitzgerald et al., 2006)
RVI	Ratio vegetation index	$\frac{NIR}{R}$	(Pearson and Miller, 1972)
OSAVI	Optimization soil-adjusted vegetation index	$\frac{NIR-R}{NIR+R+0.16}$	(Steven, 1998)
RESAVI	Red edge soil adjusted vegetation index	$\frac{1.5*\left(NIR-RE\right)}{NIR+RE+0.5}$	(Cao et al., 2013)

#### 2.5.4 Modeling methods and validation

Before constructing the AGB and LAI prediction models for rice and wheat based on the PSMICGS, the sample set was divided using a random selection method. To prevent model overfitting, we split the data for rice and wheat into modeling and validation sets at ratios of 8:2 and 7:3, respectively. Subsequently, nonlinear regression analysis was conducted to construct models to estimate the AGB and LAI of rice and wheat based on the VIs and agronomic parameters. After model construction, the models were evaluated using the coefficient of determination, R², and RMSE (Lu et al., 2021). A higher R² value closer to one and a lower RMSE indicated a better prediction performance of the model. The calculation equations are as follows:


(7) $$R^{2}=1-\frac{\sum_{i=1}^{n}{\left(y_{i}-{\hat{y}}_{i}\right)}^{2}}{\sqrt{\sum_{i=1}^{n}\left(y_{i}-{\bar{y}}_{i}\right)}},$$

where *n* is the number of samples; *y_i_* is the actual value of the ith sample; ${\hat{y}}_{i}$ is the predicted value of the ith sample; and ${\bar{y}}_{i}$ is the average value of all samples.


(8) $$\text{RMSE}=\sqrt{\frac{1}{N}\sum_{i=1}^{n}{\left(y_{i}-{\hat{y}}_{i}\right)}^{2}},$$

where *n* represents the number of samples; *y_i_* represents the actual value; and ${\hat{y}}_{i}$ represents the predicted value.

## 3 Results and discussion

This section introduces the structural assembly and calibration of the PSMICGS, along with the results of image registration. It analyzes the PSMICGS performance in terms of SNR and radiometric response accuracy testing. Furthermore, it elaborates on the results of the constructed models for estimating the AGB and LAI of rice and wheat based on the PSMICGS.

### 3.1 Analysis of the spectral image processing results based on the PSMICGS

**Figure 7:**
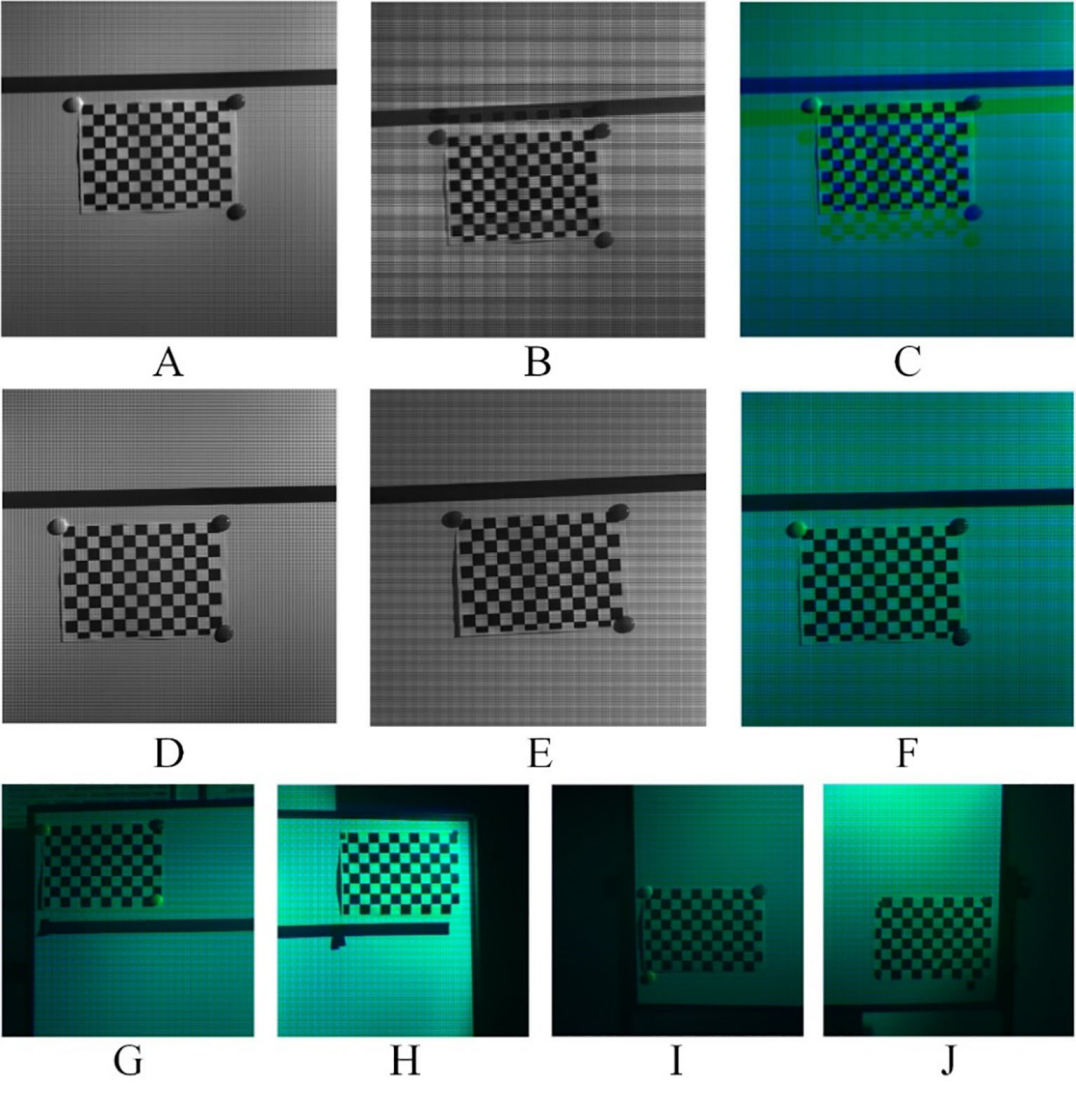
Checkerboard pattern status within the fields of view before and after the calibration of the front imaging system. Prior to calibration: **(A)** MMC1, **(B)** MMC2, **(C)** composite image of the checkerboard pattern at the middle position. Post-calibration: **(D)** MMC1, **(E)** MMC2, **(F)** composite image of the checkerboard pattern at the middle position, **(G–J)** composite images of the checkerboard pattern at the edge positions.

**Figures 7A−C** shows that prior to adjusting the front imaging system, the checkerboard pattern at the middle position of the fields of view for MMC1 and MMC2 was difficult to align accurately, resulting in noticeable misalignment. This indicated that the fields of view of the two MMCs were not in the same position. **Figures 7D−F** shows that after adjustment, the checkerboard pattern at the middle position of the fields of view for MMC1 and MMC2 achieved accurate alignments in both the horizontal and vertical directions. Further adjustment of the first and second hexagon socket set screws resulted in the alignment of the checkerboard patterns at the four edge positions, indicating that the checkerboard elements in the dual fields of view achieved alignment at the edges, as shown in **Figures 7G–J**. These adjustments successfully achieved co-optical path imaging of the dual detectors, laying the foundation for crop growth monitoring activities.

**Figure 8:**
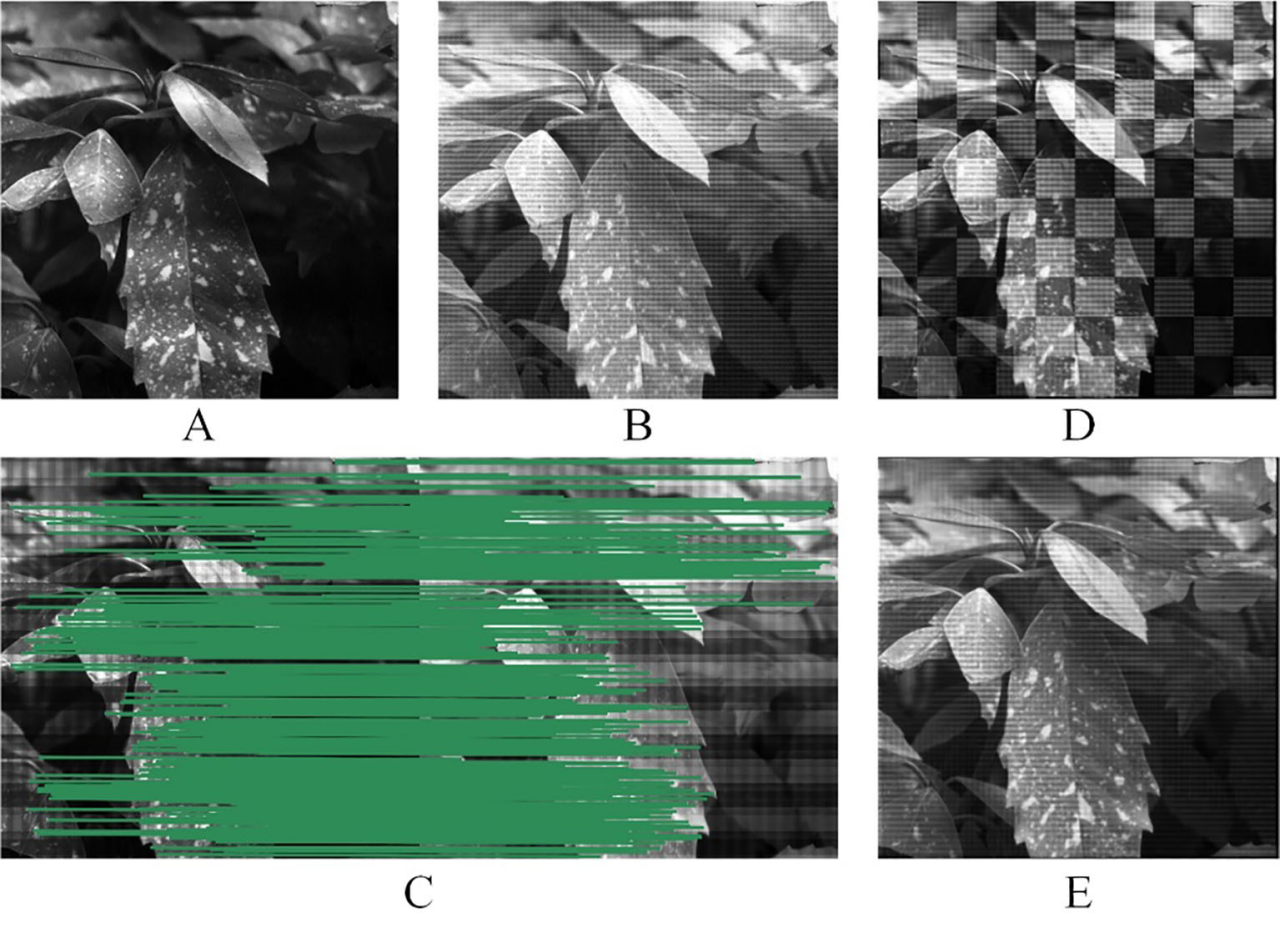
Image registration based on SIFT: **(A)** Reference image (MMC1), **(B)** image to be registered (MMC2), **(C)** feature matching graph, **(D)** mosaicked checkerboard image, and **(E)** image registration fusion graph.

To address image registration errors resulting from mechanical assembly inaccuracies, the results of image registration based on the SIFT algorithm are illustrated in **Figure 8**. **Figure 8A** shows the reference image, while **Figure 8B** shows the image to be registered, with **Figure 8B** showing the result of the MMC2 image after mirroring. During the algorithm execution phase, the nearest-neighbor distance ratio (NNDR) method identified 939 matching points, which were reduced to 340 after secondary feature point screening (FSC), as shown in **Figure 8C**. The checkerboard pattern images post-affine transformation of the dual field images, and the corresponding results are shown in **Figures 8D, E**, demonstrating precise overlap of the image regions. Following the image registration fusion, the RMSEr for the image registration was 0.5829, indicating a favorable outcome of the image registration fusion. This research underscores that the embedded control module and image registration algorithm effectively accomplished online registration of multispectral crop images.

### 3.2 Analysis of calibration results of the PSMICGS

#### 3.2.1 Analysis of the spectral calibration results of the PSMICGS

**Figure 9:**
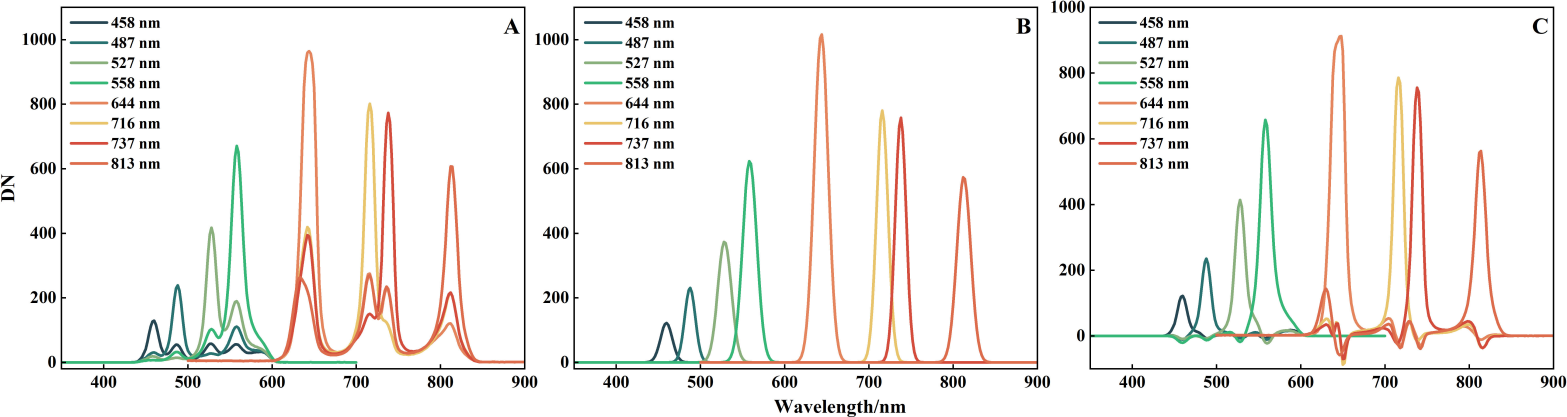
Spectral calibration results of the PSMICGS: **(A)** Original response DN curves of each channel, **(B)** Gaussian fitted response DN curves of each channel, and **(C)** DN curves after spectral crosstalk correction.

When the exposure time and gain values of the MMC1 and MMC2 were set to 100 ms and 6 dB, respectively, the maximum DN spectral channels were 558 nm and 813 nm, respectively. After adjusting the spectrometer bands in the spectral calibration system to the corresponding settings, to prevent overexposure of captured images, the maximum exposure times for MMC1 and MMC2 were 400 ms and 850 ms, respectively, when the DN values ranged between 650 and 970. Under these maximum exposure settings, we extracted and fitted the DN values from the images of each stepped spectral band according to the band arrangement in the MMC. The response DNs of each channel are shown in **Figure 9A**. The Gaussian curves of each band after further Gaussian fitting of each channel are shown in **Figure 9B**. The deviation of the central wavelength from the preset band centers remained within ±0.5 nm, with a maximum error of 0.49 nm (channel 4), as detailed in **Table 2**. This calibration result indicated that the actual central wavelengths of each channel in the PSMICGS met the requirements of the selected characteristic bands. Additionally, **Figure 9C** shows the curves after correcting the original response data using the crosstalk correction coefficient matrix. The crosstalk information between the channels was effectively corrected, and the average correlation coefficient between the corrected data for each channel and the Gaussian data exceeded 0.98.

**Table 2:** Spectral calibration results of the PSMICGS.

Channel	Theoretical central wavelength (nm)	Actual central wavelength (nm)	FWHM (nm)	Deviation (nm)
B1	458	457.60	14.12	–0.40
B2	487	486.63	12.71	–0.37
B3	527	527.41	16.15	0.41
B4	558	558.49	16.82	0.49
B5	644	644.43	14.12	0.43
B6	716	715.91	13.80	-0.09
B7	737	736.74	12.94	-0.26
B8	813	812.68	16.61	-0.32

#### 3.2.2 Analysis of the radiometric calibration results of the PSMICGS

**Figure 10:**
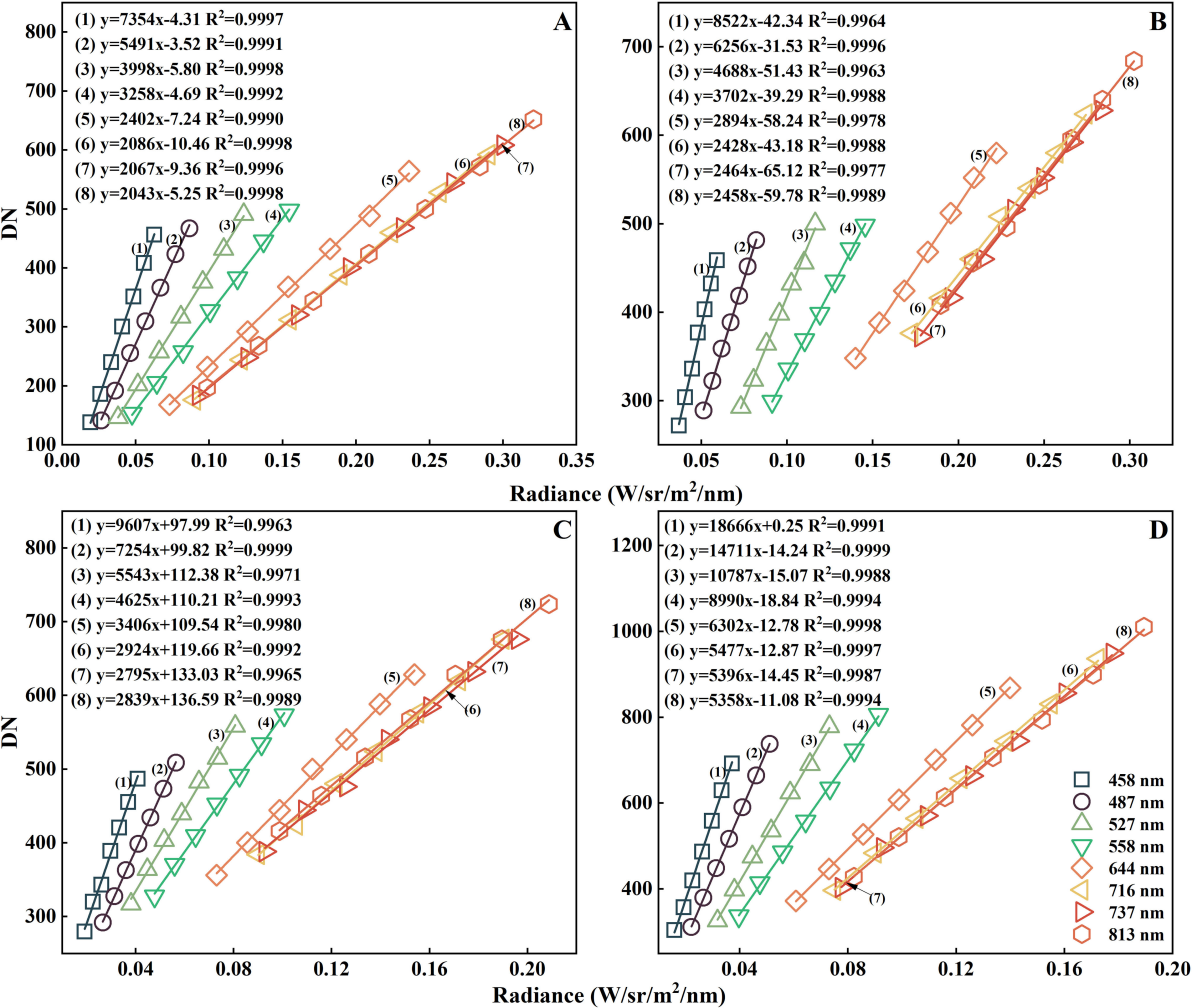
Radiometric calibration results of the PSMICGS: **(A–D)** Linear fitting graphs of the response DN of the PSMICGS and the different radiance values for exposure times set to 60, 80, 100, and 120 ms.

For the radiometric calibration of the PSMICGS, four exposure times were set (60, 80, 100, and 120 ms). The relationship curves between the response DN of the PSMICGS and the different radiance values are shown in **Figure 10**. The DN of each channel under different exposure times was derived from the average DN values of each channel in images with a uniform light source. As shown in **Figure 10**, the obtained radiance values ranged from 0 to 0.35 W/sr/m²/nm. Linear fitting of the DN and radiance at different exposure times revealed that the coefficient of determination (R²) was greater than 0.99. The calibration results indicated that the response DN of the PSMICGS had an excellent linear relationship with different radiance values at various exposure times, making it fully suitable for crop growth monitoring.

### 3.3 Analysis of the performance test results for the PSMICGS

**Figure 11:**
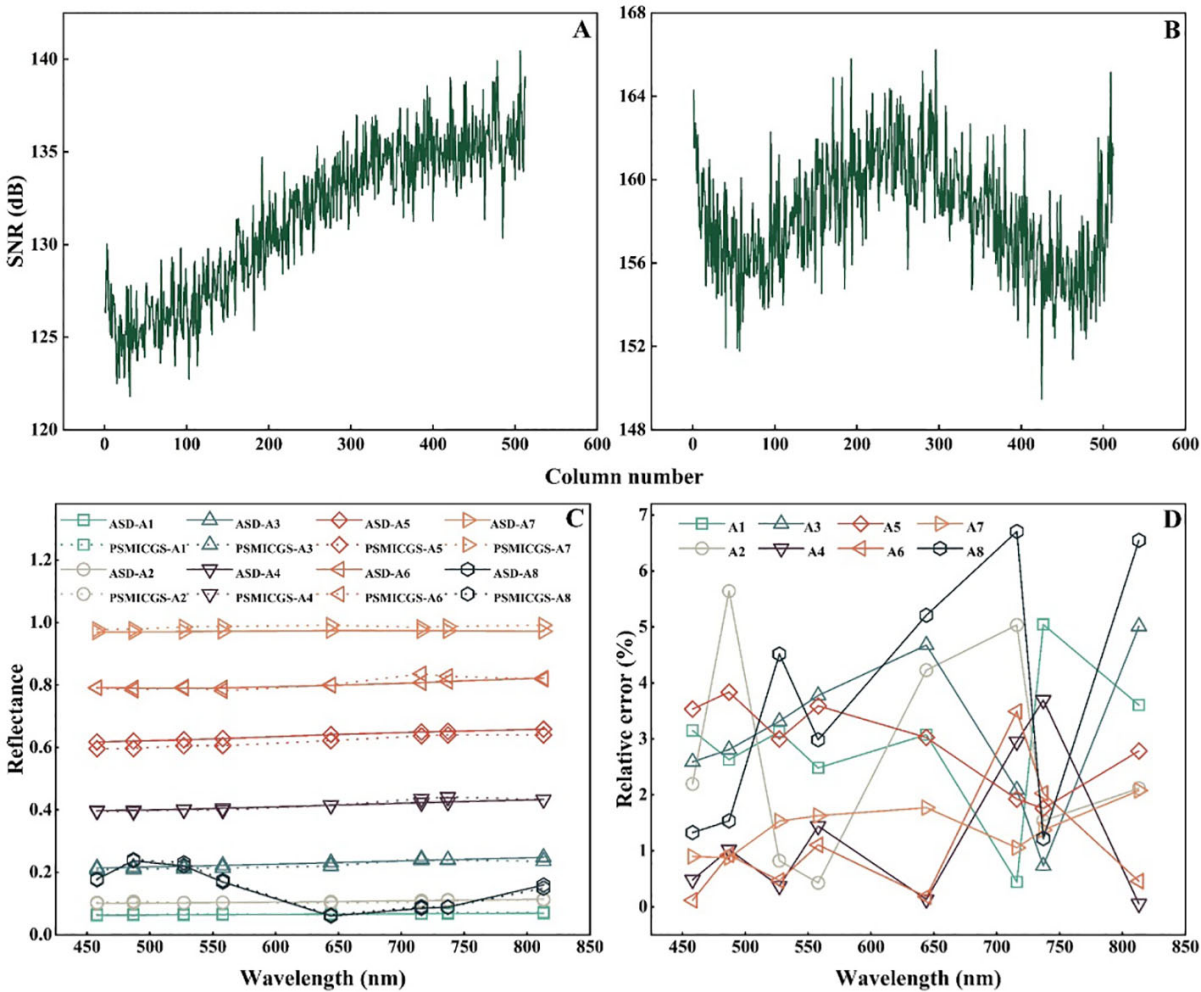
Performance test results of the PSMICGS: **(A)** Signal-to-noise ratio (SNR) statistics for MMC1, **(B)** SNR statistics for MMC2, **(C)** test values for panels A1–A8 using the PSMICGS and ASD, and **(D)** relative error values between the PSMICGS and ASD.

We analyzed the acquired images and found that the DN values for the 558 and 813 nm channels in MMC1 and MMC2 were highest when the exposure time and gain values were set to 100 ms and 6 dB, respectively. Subsequently, adjusting the exposure times to 400 for MMC1 and 850 ms for MMC2 ensured that the DN values fell within a reasonable range, establishing these settings as benchmark parameters. After setting MMC1 and MMC2 to these benchmark values, the tunable monochromatic light source imaging system was adjusted to a wavelength of 0 nm, and uniformly illuminated images were then captured. The DN values for the 558 and 813 nm channels were then extracted, and the SNRs were calculated using Equation 2. **Figures 11A, B** show that the average SNR for each column pixel exceeded 120 dB. These test results indicated that the PSMICGS exhibited excellent SNR performance, meeting the requirements for quantitative crop growth monitoring.

**Figure 11C** shows the test results for panels A1−A8 using the PSMICGS and ASD, revealing a consistent trend in the reflectance values for the different panels. The relative error values between the PSMICGS and ASD for panels with different reflectances are shown in **Figure 11D**, with relative errors within 7% across all eight bands. This demonstrates the high accuracy of the PSMICGS in terms of radiometric response.

### 3.4 Rice and wheat growth monitoring experiment based on the PSMICGS

#### 3.4.1 Sample set division

In this study, we used pre-heading data of rice and wheat to establish the prediction models for AGB and LAI. The dataset encompassed variations arising from different factors such as varieties, nitrogen levels, and planting densities. **Table 3** shows that the modeling set and validation set were partitioned using a random selection method. The modeling set exhibited significant data variability, encompassing diverse possible scenarios, suggesting that the dataset was suitable for the development of prediction models for AGB and LAI in rice and wheat.

**Table 3:** Descriptive statistics of AGB and LAI in rice and wheat.

Crop	Indicators	Sample number	Min	Max	Mean	SD
Rice	Modeled dataset					
	AGB (t/ha)	101	1.5653	14.7338	4.8396	2.3937
	LAI	101	0.1322	11.1145	1.5569	1.6120
	Validated dataset					
	AGB (t/ha)	25	2.0464	8.6708	4.2401	1.6492
	LAI	25	0.2226	4.4175	1.0977	0.9701
Wheat	Modeled dataset					
	AGB (t/ha)	133	0.3730	12.1556	4.7960	2.8263
	LAI	133	0.4220	7.9354	2.9261	1.3127
	Validated dataset					
	AGB (t/ha)	57	0.3784	11.9300	4.2322	2.2259
	LAI	57	0.4568	6.5178	2.7605	1.3502

#### 3.4.2 Relationship between AGB and LAI of rice and wheat and VI

**Figure 12:**
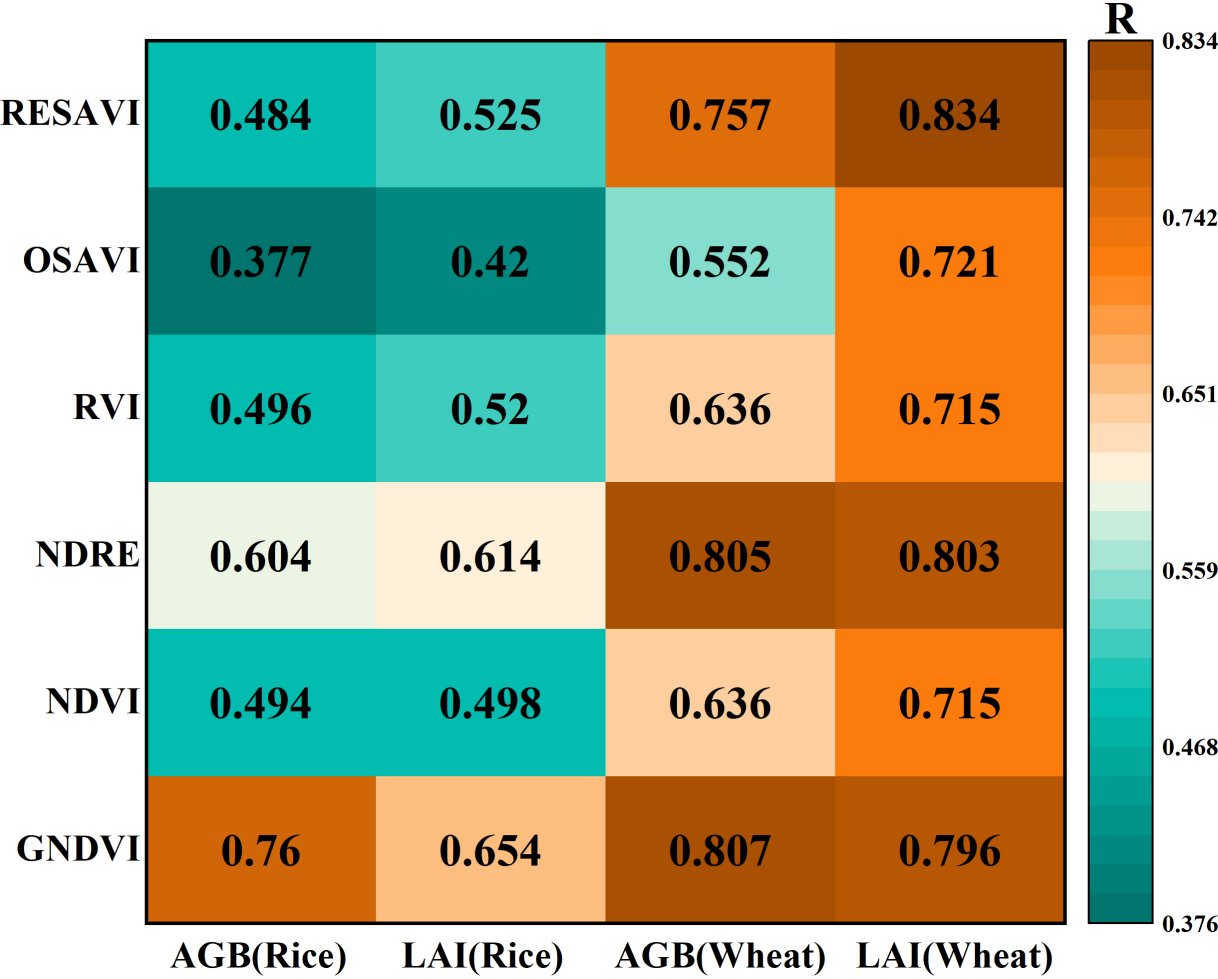
Correlation of the PSMICGS-constructed VIs with rice and wheat AGB and LAI.

To formulate prediction models for AGB and LAI in rice and wheat based on the PSMICGS, a correlation analysis between the VIs constructed using the PSMICGS and AGB and LAI was conducted. **Figure 12** shows that the VI GNDVI exhibited the highest correlation with rice AGB and LAI, with correlation coefficients (R) of 0.76 and 0.654, respectively. Additionally, the VIs, GNDVI, and RESAVI exhibited the highest correlation with wheat AGB and LAI, having correlation coefficients (R) of 0.807 and 0.834, respectively. Subsequent modeling analyses for rice and wheat AGB and LAI were conducted using the selectively optimized VIs.

#### 3.4.3 Construction of AGB and LAI monitoring model for rice and wheat based on the PSMICGS

**Figure 13:**
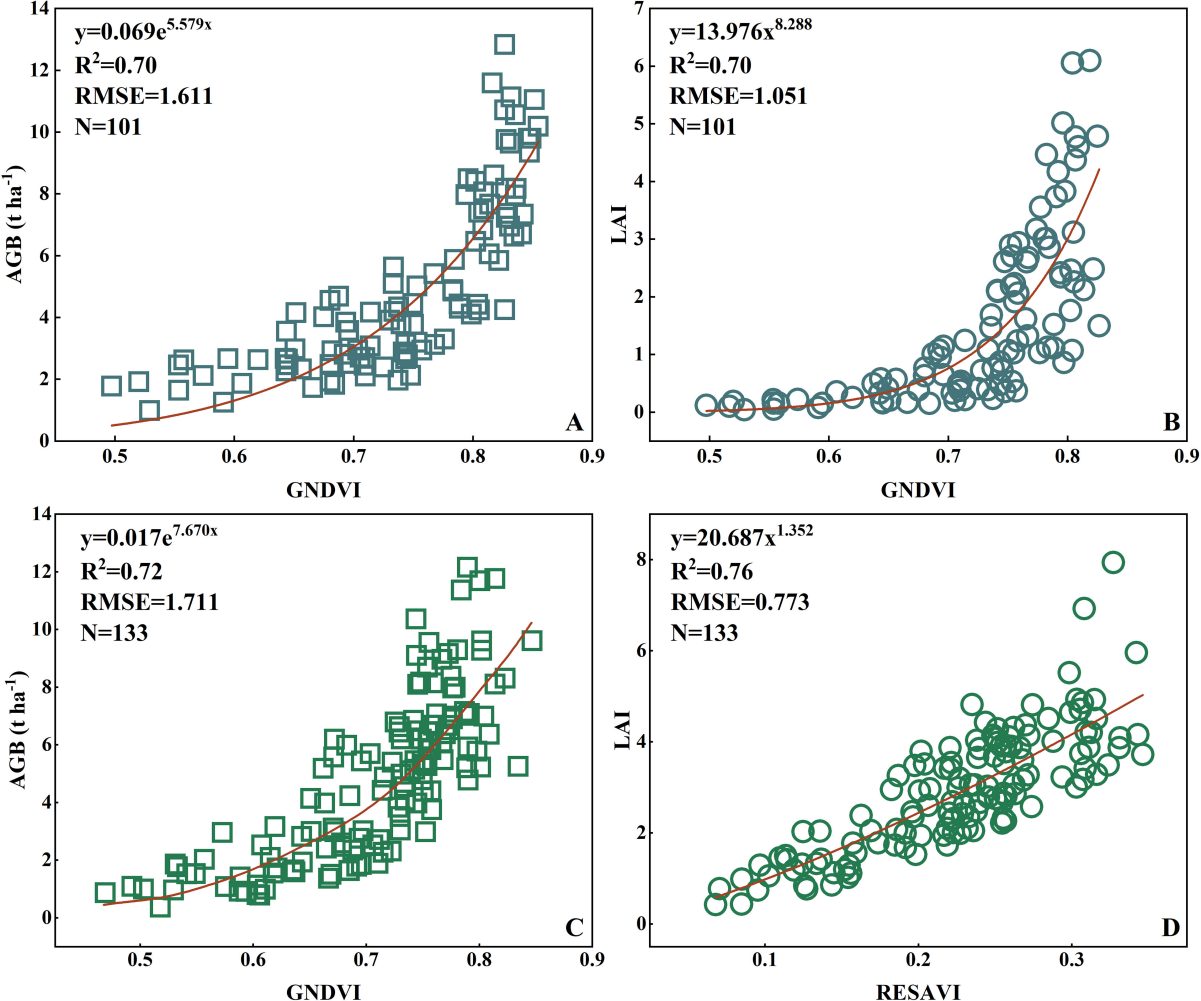
Prediction models of the rice and wheat AGB and LAI constructed based on the PSMICGS. **(A, B)** are rice AGB and LAI, respectively, and **(C, D)** are wheat AGB and LAI, respectively.

Based on the selected VIs, prediction models for AGB and LAI in rice and wheat were established. **Figures 13A, B** show the models for rice AGB and LAI developed using the VI GNDVI, resulting in determination coefficients R² of 0.7 and RMSE values of 1.611 t/ha and 1.051, respectively. For wheat (**Figures 13C, D**), the AGB and LAI prediction models were constructed using the VIs GNDVI and RESAVI, yielding R² values of 0.72 and 0.76, respectively, with corresponding RMSE values of 1.711 t/ha and 0.773, respectively. In summary, the prediction models for AGB and LAI in rice and wheat constructed based on the PSMICGS demonstrated satisfactory performance.

#### 3.4.4 Validation of the AGB and LAI monitoring model for rice and wheat based on the PSMICGS

**Figure 14:**
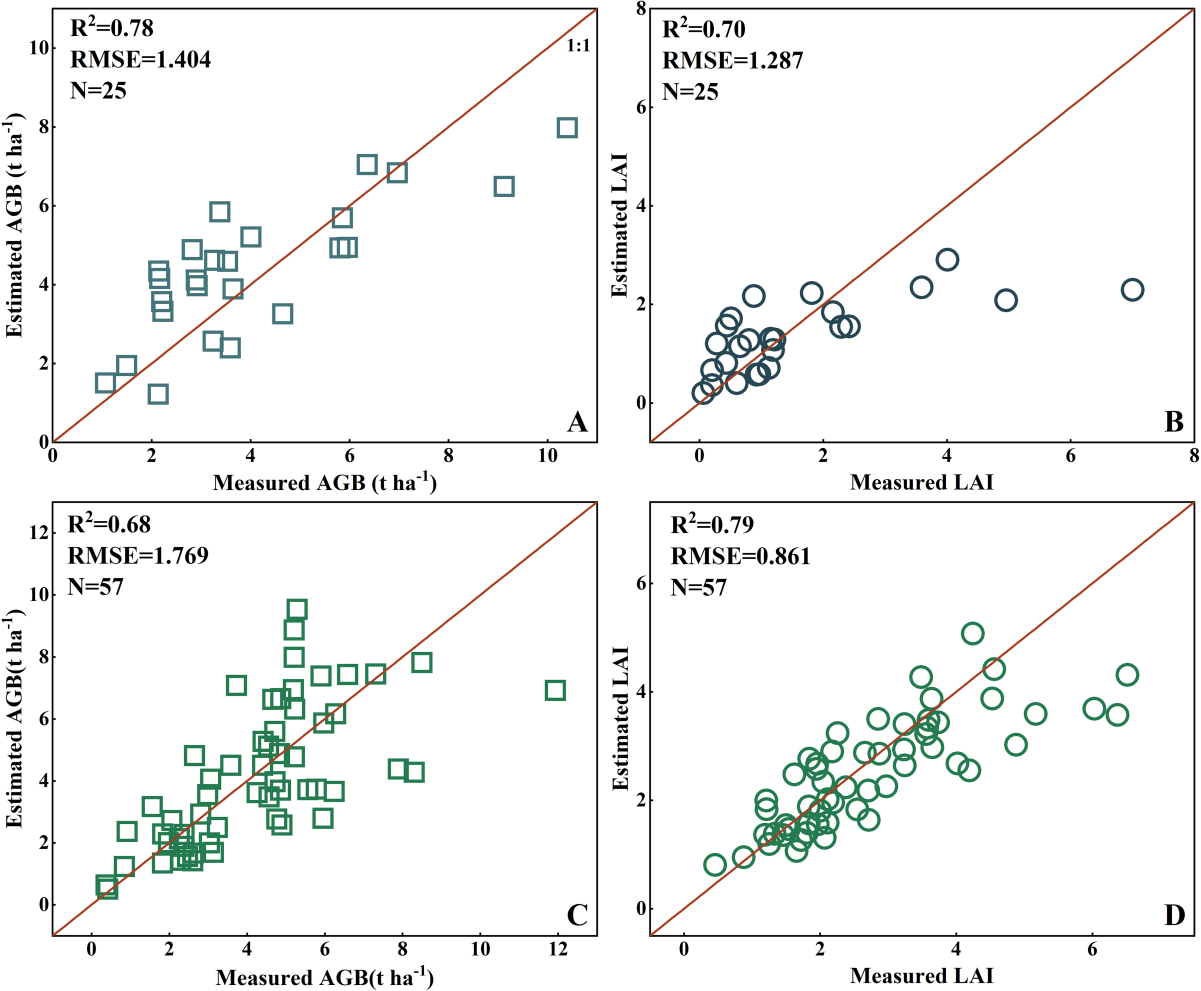
Scatter plots for the validation of the prediction models of rice and wheat AGB and LAI constructed based on the PSMICGS: **(A, B)** are the rice AGB and LAI, respectively, and **(C, D)** are the wheat AGB and LAI, respectively.

The constructed prediction models for the AGB and LAI in rice and wheat were validated using the validation dataset. **Figure 14** shows that the validation determination coefficients, R², for the rice AGB and LAI estimation models based on the PSMICGS were 0.78 and 0.70, respectively, with RMSE values of 1.404 t/ha and 1.287, respectively. For the wheat AGB and LAI estimation models, the validation R² values were 0.68 and 0.79, respectively, with corresponding RMSE values of 1.769 t/ha and 0.861, respectively. Overall, the validation results for the constructed AGB and LAI models in rice and wheat were favorable, indicating the feasibility of the developed estimation models.

## 4 Discussion

Portable spectral imaging devices play crucial roles in real-time and non-destructive crop growth monitoring at the field scale. However, many commercially available sensors do not provide direct output of crop growth parameters (Jia et al., 2019; Shao et al., 2023). Customized devices often lack specific bands tailored for crop growth characteristic, limiting their effectiveness (Tang et al., 2022; Wang et al., 2022; Wang et al., 2020a). To address these challenges and capitalize on the spectral sensing mechanism in crop growth, we developed the PSMICGS using MF spectrometry with a wide band range. This device allows for real-time online acquisition and interpretation of crop spectrum information. However, due to constraints in band settings and associated processes, achieving optimal characteristic bands using a single MF was challenging. Thus, further exploration and refinement of these processes are required.

The online processing of spectral information lays the foundation for real-time crop growth interpretation. By leveraging the structural features of the PSMICGS, we introduced mechanical adjustments and a multi-spectral image registration method using the SIFT algorithm to achieve precise registration of dual-field images and acquire comprehensive crop spectrum data. However, our study on SIFT-based image registration underscored the need for further exploration into optimizing image registration methods tailored to different crops and varying collection heights, especially for monitoring diverse crop growth characteristics.

Real-time interpretation of crop growth information provides reference data for crop growth diagnosis. We constructed estimation models for rice and wheat AGB and LAI based on the PSMICGS and successfully completed the entire data collection and analysis process, from crop spectrum acquisition to growth interpretation. However, the overall accuracy of the constructed models still requires further improvement. Future research should focus on estimating different ecological points and additional agronomic parameters, particularly in complex field environments. Exploring algorithms to remove water and soil background effects will be essential for improving prediction accuracy and stability. Moreover, extending crop growth monitoring studies to include different crops such as soybeans, oilseed rape, and maize will improve the applicability of the PSMICGS.

## 5 Conclusion

In this study, we developed a novel PSMICGS based on crop spectral sensing mechanisms utilizing MFs. The design included a front imaging system utilizing DMs for spectral splitting, and it used wide-band integrated co-optical path imaging to acquire crop spectral images across a broad range. We explored mechanical adjustment methods for the wide-band range front imaging system and developed image registration fusion algorithms, enhancing the precision of multispectral image registration fusion for crops. Additionally, we integrated sensor information with crop growth monitoring models, enabling the real-time interpretation of multiple agronomic features. Performance tests demonstrated that the device achieved a good SNR (>120 dB) and accurate radiometric response (relative error < 7%). Growth monitoring experiments for rice and wheat validated the prediction models for AGB and LAI and achieved determination coefficients (R²) greater than 0.7, indicating that the models had good prediction accuracy. In summary, this research provides a foundational tool for monitoring crop organs and canopies, with potential applications in advancing agricultural production efficiency.

## Data Availability

The original contributions presented in the study are included in the article/**Supplementary Material**. Further inquiries can be directed to the corresponding authors.
