# Supplementary material for: Design and implementation of a portable snapshot multispectral imaging crop-growth sensor
Source: Front Plant Sci. 2024 Aug 26;15:1416221. doi: 10.3389/fpls.2024.1416221 (PMC11381302; doi:10.3389/fpls.2024.1416221)
Supplement: Supplementary file 1 [file Table1.docx]

**Table 1** MMC1 crosstalk correction coefficient matrix

| j\i | P_1_ | P_2_ | P_3_ | P_4_ |
| --- | --- | --- | --- | --- |
| PO_1_ | 1.0261 | -0.2538 | -0.1388 | -0.0149 |
| PO_2_ | -0.3290 | 1.0816 | -0.0006 | -0.1662 |
| PO_3_ | -0.2097 | -0.0073 | 1.1013 | -0.3227 |
| PO_4_ | -0.1400 | -0.1507 | -0.2853 | 1.0980 |

**Table 2** MMC2 crosstalk correction coefficient matrix

| j\i | P_5_ | P_6_ | P_7_ | P_8_ |
| --- | --- | --- | --- | --- |
| PO_5_ | 1.2157 | -0.3734 | -0.3003 | -0.0182 |
| PO_6_ | -0.4276 | 1.2604 | 0.0417 | -0.4181 |
| PO_7_ | -0.3665 | 0.0076 | 1.1999 | -0.3956 |
| PO_8_ | -0.0220 | -0.3603 | -0.3055 | 1.1582 |
